# Optimization of *Bacillus amyloliquefaciens* BLB369 Culture Medium by Response Surface Methodology for Low Cost Production of Antifungal Activity

**DOI:** 10.3390/microorganisms10040830

**Published:** 2022-04-16

**Authors:** Imen Zalila-Kolsi, Sameh Kessentini, Slim Tounsi, Kaïs Jamoussi

**Affiliations:** 1 Laboratory of Biopesticides, Centre of Biotechnology of Sfax, University of Sfax, P.O. Box 1177, Sfax 3018, Tunisia; slim.tounsi@cbs.rnrt.tn (S.T.); kaisjamoussi8@yahoo.fr (K.J.); 2 Department of Health and Medical Sciences, Khawarizmi International College, Abu Dhabi P.O. Box 25669, United Arab Emirates; 3 Laboratory of Probability and Statistics, Faculty of Sciences of Sfax, University of Sfax, P.O. Box 1171, Sfax 3000, Tunisia; samehkessentini@gmail.com

**Keywords:** antifungal activity, response surface methodology, Plackett–Burman design, central composite design, polynomial and trigonometric regression models

## Abstract

*Bacillus amyloliquefaciens* BLB369 is an important plant growth-promoting bacterium, which produces antifungal compounds. A statistics-based experimental design was used to optimize a liquid culture medium using inexpensive substrates for increasing its antifungal activity. A Plackett–Burman design was first applied to elucidate medium components having significant effects on antifungal production. Then the steepest ascent method was employed to approach the experimental design space, followed by an application of central composite design. Three factors were retained (candy waste, peptone, and sodium chloride), and polynomial and original trigonometric models fitted the antifungal activity. The trigonometric model ensured a better fit. The contour and surface plots showed concentric increasing levels pointing out an optimized activity. Hence, the polynomial and trigonometric models showed a maximal antifungal activity of 251.9 (AU/mL) and 255.5 (AU/mL) for (19.17, 19.88, 3.75) (g/L) and (19.61, 20, 3.7) (g/L) of candy waste, peptone, and NaCl, respectively. This study provides a potential strategy for improving the fermentation of *B. amyloliquefaciens* BLB369 in low-cost media for large-scale industrial production.

## 1. Introduction

Phytopathogenic fungi cause several plant diseases responsible for various crop losses and agricultural products deterioration. *Fusarium graminearum* produces various pathogenicity and virulence factors allowing it to enter into the plant and advance within the interior of the infected tissue. It causes Fusarium head blight (FHB), one of the most economically disastrous diseases of wheat, barley, rice, and other grain crops worldwide [1,2]. It produces deoxynivalenol (DON) and zearalenone, two harmful toxins to humans and animals. Consequently, chemical fungicides were used. Triazoles (e.g., tebuconazole, metconazole, and prothioconazole [3,4]) and benzimidazole obstruct sterol biosynthesis and are the most effective to suppress FHB symptoms and decrease mycotoxin concentration [5,6,7]. Prochloraz, an imidazole derivate acting similarly to triazole fungicides [8], is widely applied to control fungal growth in cereals in several European countries [9]. The widespread use of chemical pesticides (more than 97% of control measures) overexposed nature to their ecotoxicity and led to a loss of efficiency due to adaptation of the targeted phytopathogens and disadvantages on non-target populations sharing the ecosystem [10]. High doses of triazole fungicides strongly affect the structure of the microbial communities in soil and usually decrease the soil microbial population and the activities of enzymes found in soil [11]. The use of non-pathogenic microorganisms as biopesticides is an emerging technology, is ecologically compatible, and is considered a promising alternative to synthetic pesticides [12,13,14]. Numerous *Bacillus* species offer many advantages for agricultural biotechnology such as *Bacillus amyloliquefaciens*, *B. subtilis*, *B. licheniformis*, and *B. pumilus*. They synthesize several secondary metabolites, essentially, the cyclic lipopeptides surfactin, iturin, and fengycin with antifungal activities [15,16,17,18]. They also produce primary metabolites with antifungal activities like the hydrolytic enzymes (chitinase, glucanase, and protease enzymes) acting on fungal cell walls [19,20,21]. The growth of cells and metabolite concentrations is influenced strongly by medium composition such as the carbon source, nitrogen source, and inorganic salts. Efforts must therefore be redirected to improve production efficiency and recovery bioprocesses to optimize yields [22,23].

The statistical methods using screening and response surface methodology or artificial neural networks offer several advantages over conventional methods to optimize numerous multi-factorial processes or formulations [24,25,26,27,28]. They shortlist significant nutrients for culture media, help understand the interactions among the nutrients at various concentrations, and reduce the total number of experiments leading to saving time and resources [29,30,31]. They allow the production increase of antagonist compounds, spores, and enzymes by *Bacillus* spp. strains [32,33,34,35]. Many researchers have used central composite design (CCD) to identify optimal reaction conditions [36,37]. Therefore, the present study was undertaken to optimize a medium for economical production of *B. amyloliquefaciens* BLB369 antifungal activity. We applied a Plackett–Burman design (PBD) to screen the significant factors, the steepest ascent method (SAM) to approach the experimental design space, and the (CCD) to optimize the concentrations of selected variables using two different regression models.

## 2. Materials and Methods

### 2.1. Microorganisms and Cultivation

The *B. amyloliquefaciens* BLB369 strain was previously characterized by Zalila-Kolsi et al. [38]. In fact, it was isolated in our laboratory from Tunisian rhizosphere soil sample and then identified by using API50CH and API20E strips and partially sequencing 16S rDNA and *gyrA* genes. It could produce the extracellular cyclic lipopeptides iturin and surfactin harboring antifungal activities. Its antagonist effect against *F. graminearum* for protection of durum wheat was demonstrated in vivo [38]. The phytopathogenic fungus *Fusarium graminearum* was kindly provided by the Agricultural Culture Collection of Biopesticides laboratory, Centre of Biotechnology of Sfax. It was maintained on potato dextrose agar (PDA) and used as the target pathogen for testing antifungal activity. For spore suspension preparation, the fungus was incubated on a PDA plate for 5 days at 28 °C; then, 0.9% NaCl solution was added, and the top of the mold was scraped with a sterile loop to release spores. The collected fungus suspension was filtered with sterile cotton to remove mycelial fragments. The obtained spore suspension was enumerated on Malassez cell, adjusted to a concentration of approximately 10^5^ spores/mL, and stored at 4 °C.

The *B. amyloliquefaciens* BLB369 was grown in a 250 mL flask containing 50 mL of MOLB medium [39] at 30 °C for 14 h, on a rotary shaker set 200 rpm in order to prepare the culture inoculums. For antifungal production, the inoculums were served to inoculate 50 mL of culture medium in a 250 mL flask with an initial OD_600_ of 0.15, and the culture was incubated at 30 °C for 48 h, shaking at 200 rpm.

### 2.2. Determination of the Antifungal Activity

The antagonistic activity of *B. amyloliquefaciens* BLB369 strain against *F. graminearum* was assessed by the well diffusion method. The BLB369 culture broth of 48 h was centrifuged in 2 mL Eppendorf tube at 10,000 rpm for 15 min, and the culture medium was recovered. Several dilutions of the bacterial culture medium were prepared in the MOLB medium with various dilution factors (DF) (values: 1, 2, 3, 4, 5, …, 30) and used for antifungal activity determination. The dilution factor may be expressed as the ratio of the volume of the final diluted bacterial culture medium to the initial volume removed from the original bacterial culture medium. Then, aliquots (v = 100 µL) were filled in wells of 5 mm diameter made in PDA + chloramphenicol (30 µg/mL) pH 7.0 previously plated with 100 µL of collected *F. graminearum* suspension (10^5^ spores/mL). After incubation at 28 °C for 3–5 days, the inhibition zones were observed. Antifungal activity (AU/mL) = (DF^h^ * 1000/v), with AU: Arbitrary Unit, DF^h^: higher dilution factor of the bacterial culture medium able to inhibit fungal growth. v: volume (µL) of the diluted bacterial culture medium used for well test. Each experience was repeated three times.

### 2.3. Identification of Significant Factors Using Plackett–Burman Design

The PBD [40] was used to select the most significant factors of medium components for biofungicides production where the interactions between the factors were considered negligible. An orthogonal matrix was generated using seven factors, and each factor was represented by a high level (+1) and a low level (−1). Carbon sources, nitrogen sources, and salts could influence the production of antifungal activity. The candy waste was an agro-industrial by-product corresponding to an aqueous solution rich in carbohydrates. Its reducing sugar content was estimated to be about 56.5 g/L using the colorimetric method with 3.5-dinitrosalicylic acid reagent (*DNS*) [41]. The fish extract was obtained from tuna canning industry waste after being treated with NaOH solution at pH 10-12 for 2 h. Its nitrogen content (non-protein nitrogen and nitrogen in proteins) was estimated to be about 40 g/L using the Kjeldahl method. Yeast extract is essentially composed of amino acids, peptides, carbohydrates, and soluble vitamins. Peptone from casein enzymatic digest is a rich source of peptides and amino acids. Hence, the candy waste expressed as reducing sugar (g/L) (10, 20), fish extract expressed as nitrogen content (g/L) (0, 16), peptone from casein enzymatic digest (Fluka) (g/L) (0, 10), yeast extract (Sigma-Aldrich) (g/L) (0, 5), NaCl (g/L) (0, 4), MgSO_4_ (g/L) (0, 0.5), and MnSO_4_ (g/L) (0, 0.006) corresponding to (−1, +1) levels were screened for biofungicides production. Each of the 8 experiences reported in the PBD is repeated twice (Table 1). A linear approach presented by the following equation (Equation (1)) was considered to be sufficient for screening:

(1) $$Y_{1}=\alpha_{0}+\alpha_{1}\;A+\alpha_{2}\;B+\alpha_{3}\;C+\alpha_{4}\;D+\alpha_{5}\;E+\alpha_{6}\;F+\alpha_{7}\;G$$

where *Y_1_* is the predicted target response (the antifungal activity); *α_i_* are the regression coefficients; and *A*, *B*, *C*, *D*, *E*, *F*, and *G* are dimensionless coded values of the independent variables candy waste, fish extract, peptone, yeast extract, NaCl, MgSO_4_, and MnSO_4_, respectively. The experimental data were fitted using MATLAB software. The most significant factors were then investigated more thoroughly in subsequent experiments (Table 2).

### 2.4. Optimization by Steepest Ascent Method

The path of the SAM was achieved to set up basal concentrations of media components selected from the PBD to be used in a CCD. It permitted rapid movement towards the most favorable of variable concentrations. Increments are direct ratios of regression coefficients *α_i_* (Equation (1)). Experiments were performed along with the SAM until the response did not increase anymore, and the starting point was the center of PBD, i.e., the medium level of factors reported in Table 1 and Table 3.

### 2.5. Central Composite Design and Response Surface

#### CCD Matrix and Antifungal Activity

Once the critical factors were identified via screening and the experimental design space was approached by SAM, the CCD was used to define the level of the significant parameters and the interactions between them, which significantly influence the antifungal activity. Each parameter was analyzed at five levels coded as (−2, −1, 0, +1, +2) (Table 4).

### 2.6. Regression Models and Statistical Analysis

The experimental data were fitted using MATLAB and Eureqa software. To determine the antifungal activity relation to input variables according to Table 4, a polynomial regression and then a trigonometric model were used. The second-order polynomial regression was first applied:

(2) $$Y_{1}=\beta_{0}+\overset{3}{\underset{i=1}{\sum }}\beta_{i}X_{i}+\overset{3}{\underset{i<j=2}{\sum }}\beta_{ij}X_{i}X_{j}+\overset{3}{\underset{i=1}{\sum }}\beta_{ii}X_{i}^{2}$$

where *Y_1_* is the antifungal activity; *X_i_* are input variables (3 variables retained); $\beta_{0}$, $\beta_{i}$, $\beta_{ij}$, and $\beta_{ii}$ are, respectively, the regression coefficients for the intercept, linear, interaction, and quadratic effects. The model was then adjusted using the stepwise technique [42]. Given that the three retained variables took limited levels denoted by $\;{\bar{X}}_{1}$,${\bar{\;X}}_{2}$, and ${\bar{X}}_{3}$, an alternative trigonometric model with oscillating behavior was investigated. For a limited number of regression coefficients, we considered the following trigonometric model optimized by Eureqa software:

(3) $$Y_{1}=a+b\;\cos\left({\bar{X}}_{2}\right)\;\cos\left({\bar{X}}_{3}\right)+\;c\;\cos\left(d-e{\bar{X}}_{1}\right)$$

We were motivated by finding the model that guarantees high values of the coefficient of determination (*R^2^*), the adjusted *R^2^* (*AR^2^*), and the corrected Akaike information criterion (*AICc*). The *R^2^* increases every time we add predictors, even those insignificant. Therefore, the *AR^2^* was introduced to compare the explanatory power of regression models and was correlated to *R^2^* [43]. The *AR^2^* increases only if the additional term is a good predictor, and it decreases with poor quality predictors. The Akaike information criterion rewards goodness of fit (using the likelihood); however, it penalizes increasing the number of estimated coefficients, which may differ from the number of predictors [44]. The *AICc* was introduced by Hurvich and Tsai [45] as a correction to the Akaike information criterion for small samples.

## 3. Results and Discussion

### 3.1. Screening of the Significant Medium Components Using PBD

The PBD was used to screen the seven factors candy waste, fish extract, peptone, yeast extract, NaCl, MgSO_4_, and MnSO_4_ with main effects on antifungal activity production by *B. amyloliquefaciens* BLB369 expressed in arbitrary unit per milliliter (AU/mL) (Table 1). The magnitudes and signs of each effect of the variables were shown. Candy waste (*A*), peptone (*C*), and NaCl (*E*) were the main variables that positively affected antifungal activity production as their *p*-values were lower than 1‰ (Table 2). The obtained linear model is given by

*Y_1_* = 101.563 + 17.188 *A* + 48.438 *C* + 14.063 *E* (4)

The peptone was the most significant factor due to its pronounced coefficient effect. It was manufactured by controlled enzymatic hydrolysis of casein, which is an excellent organic nitrogen source necessary for the bacteria to synthesize proteins and nucleic acids. Additionally, the industrial by-product candy waste constitutes a good carbon source for the growth of BLB369 strain and could then be valued for antifungal production. NaCl impacts the antifungal activity. It constitutes a nutriment for bacteria and has an osmotic effect in the culture medium. It could affect enzymatic activities and bacterial growth as it influences water and salts transport across bacterial membrane and could affect secretion and stability of the antifungal compounds. The fish extract did not affect the antifungal activity, probably due to fish extract instability. Therefore, the fish extract, yeast extract, MgSO_4_, and MnSO_4_ not retained after PBD screening were not added to the media in the coming runs as their concentrations at lower levels (−1) correspond to zero. The steepest ascent method further investigated the significant factors to optimize the experimental design space.

### 3.2. Optimization by Steepest Ascent Method

The direction of the steepest ascent method was determined by Equation (4). Concentrations of candy waste, peptone, and NaCl were increased because they presented positive effects on antifungal production. Hence, increments were direct ratios to regression coefficients *α_i_*, corresponding to 0.354 and 0.289 units of coded variables $\;{\bar{X}}_{1}$ and ${\bar{X}}_{3}$ for each unit of ${\bar{X}}_{2}$, respectively. When the concentrations of candy waste, peptone, and NaCl were 20.25, 20, and 3.74 (g/L), respectively, the production of antifungal activity reached its maximal value of 250 (AU/mL) at run number 4 (Table 3). This point was chosen as a clue to set up basal concentrations for further optimization by CCD (the center point for optimization by CCD).

**Table 3:** Experimental design and response of the SAM experiments.

Run	Candy Waste		Peptone		NaCl		Antifungal Activity (AU/mL)
	${\bar{X}}_{1}$ 0.354 ^#^	*X_1_* (g/L) 1.77 ^#^	${\bar{X}}_{2}$ 1 ^#^	*X_2_* (g/L) 5 ^#^	${\bar{X}}_{3}$ 0.289 ^#^	*X_3_* (g/L) 1.58 ^#^	
**1**	0	15.00	0	5	0	2.00	100
**2**	0.354	16.77	1	10	0.289	2.58	100
**3**	0.710	18.55	2	15	0.578	3.16	175
**4**	1.065	20.25	3	20	0.867	3.74	250
**5**	1.420	22.00	4	25	1.156	4.30	250
**6**	1.775	23.75	5	30	1.445	4.90	225

${\bar{X}}_{i}$: variables in coded levels; *X_i_*: variables in real values; ^#^: increment.

### 3.3. Optimization of the Selected Medium Components Using the CCD

The CCD was used to define the optimum levels of the significant factors and study their interactions. The candy waste, peptone, and NaCl (denoted in what follows by *X_1_, X_2_, X_3_*, respectively) were studied at five levels (−2, −1, 0, +1, +2). The experimental design and the experimental responses of antifungal activity (*Y_1_*) were reported (Table 4).

**Table 4:** Response surface of CCD and results for antifungal activity.

Run	Candy Waste		Peptone		NaCl		Antifungal Activity
	${\bar{X}}_{1}$	*X_1_* (g/L)	${\bar{X}}_{2}$	*X_2_* (g/L)	${\bar{X}}_{3}$	*X_3_* (g/L)	*Y_1_* (AU/mL)
**1**	+1	22	−1	15	+1	4.3	175
**2**	−1	18.4	+1	25	+1	4.3	150
**3**	−1	18.4	−1	15	−1	3.1	175
**4**	0	20.2	0	20	0	3.7	250
**5**	0	20.2	0	20	0	3.7	250
**6**	+1	22	+1	25	−1	3.1	150
**7**	−1	18.4	−1	15	+1	4.3	175
**8**	+1	22	+1	25	+1	4.3	150
**9**	−1	18.4	+1	25	−1	3.1	175
**10**	0	20.2	0	20	0	3.7	250
**11**	+1	22	−1	15	−1	3.1	125
**12**	0	20.2	0	20	0	3.7	250
**13**	0	20.2	0	20	−2	2.5	125
**14**	0	20.2	0	20	0	3.7	250
**15**	+2	23.8	0	20	0	3.7	250
**16**	0	20.2	+2	30	0	3.7	125
**17**	−2	16.6	0	20	0	3.7	200
**18**	0	20.2	0	20	0	3.7	250
**19**	0	20.2	0	20	+2	4.9	125
**20**	0	20.2	−2	10	0	3.7	125

#### 3.3.1. Regression Models for Antifungal Activity and Their Comparison

The regression coefficients were calculated using the least square technique and reported in Table 5. The standard errors and *p*-values were also reported. The standard error of the coefficient indicates the precision of the coefficient estimates. The *p*-value indicates the significance of each coefficient (low *p*-values indicate statistically significant terms). The significant variables are highlighted by asterisks: those with *p*-values less than 0.05 (Table 5). Moreover, to compare the resulting models, *R^2^*, *AR^2^*, *AICc*, and *F*-value for regression model and lack of fit are reported (Table 5 and Table 6).

##### Polynomial Regression

The polynomial fit shows only five significant terms (Table 5). However, before postulating such a statement, the model should be adjusted using the stepwise technique [42]: adding or removing variables one by one based on their *p*-value (the term with the highest *p*-value must be removed first). As additional terms, we tested including cubic (e.g., *X_1_^3^*) and second-order interaction terms (e.g., *X_1_^2^X_2_*). The retained polynomial model is given by the following equation (Table 5):

(5) $$Y_{1}=-12312+1624X_{1}+53X_{2}+351X_{3}-78X_{1}^{2}-1.33X_{2}^{2}+1.25X_{1}^{3}-8.3X_{3}^{3}$$

Comparing results before and after applying the stepwise technique (Table 5), although a lower number of predictors (8 versus 10), the coefficient of determination is slightly improved after applying the stepwise technique from 0.92 to 0.928. The *AR^2^* increased from 0.848 to 0.887, indicating more suitable predictors. The *AICc* criterion gets also improved (a lower value is observed of 190 versus 208). The *F*-value of the retained polynomial model was also computed (Table 6) and evaluated to 22, with a low *p*-value exhibiting the significance of the model. However, the hypothesis of lack of fit could not be rejected because of the high *F*-value associated with the lack of fit. This result is coherent with the relatively low *AR^2^*, and an alternative regression model should be examined.

##### Trigonometric Model

We started by estimating the coefficients through iterative optimization process. Then, we carried linear regression to check the coefficients of all terms and predict their significance (Table 5). The resulting equation is

(6) $$Y_{1}=145+87\;\cos\left({\bar{X}}_{2}\right)\;\cos\left({\bar{X}}_{3}\right)+23.8\;\cos\left(5.5-2.3{\bar{X}}_{1}\right)$$

The trigonometric model guarantees satisfactory values of both *R^2^* and *AR^2^* around 0.96 (96% of the variability in the response could be explained by this model), which are better than those obtained with the polynomial fit. The *AICc* criterion also shows the superiority of the trigonometric model with the lowest value of 160, which means a good compromise between the model fit and slight complexity. Moreover, the trigonometric model guarantees the lowest mean absolute error and root mean squared error showing a good agreement between the experimental and predicted values (Table 5). Finally, the ANOVA results demonstrate that the model is significant with an *F*-value of 212 and an associated *p*-value less than 10^−12^. The *p*-value associated with the lack of fit is equal to 0.14, indicating that the lack of fit is not significantly associated with the pure error (Table 6). All these statistical parameters illustrate the adequacy of the trigonometric model.

##### Predicted Versus Actual Plot and Residuals Versus Fits Plot

**Figure 1:**
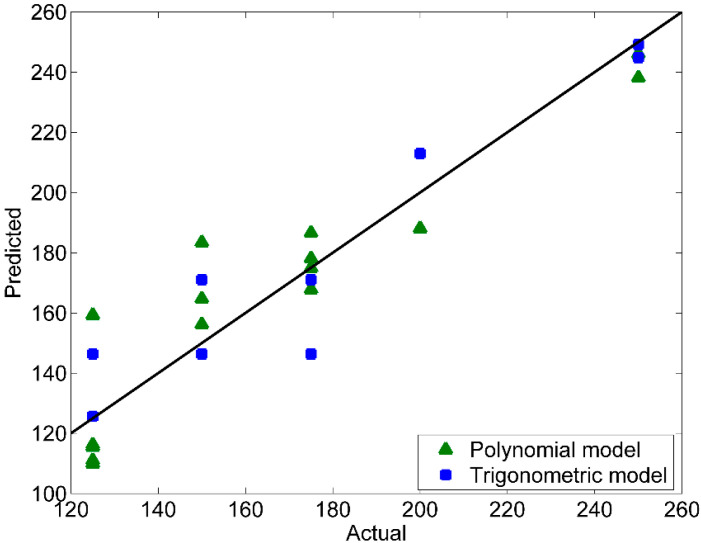
Plot of the actual versus predicted values.

**Figure 2:**
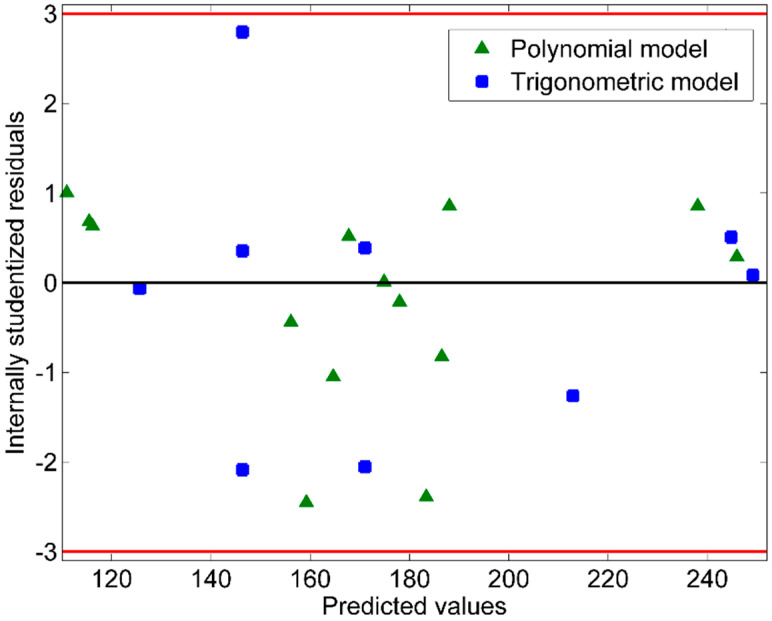
Plot of the internally studentized residuals versus the predicted values.

Figure 1 displays the predicted values by the retained polynomial and the trigonometric models, calculated from Equations (5) and (6), as a function of the actual values. Most markers (triangles for the polynomial model and squares for the trigonometric model) are scattered near the first bisector. This indicates that the predicted values are in good agreement with the actual ones. Figure 2 presents the internally studentized residuals versus the predicted values. All values are in theinterval (−3; +3) and almost equally scatter above and below the *x*-axis. Hence, both models produce random residuals, which means they are unbiased models.

##### Response Surface and Contour Plots

**Figure 3:**
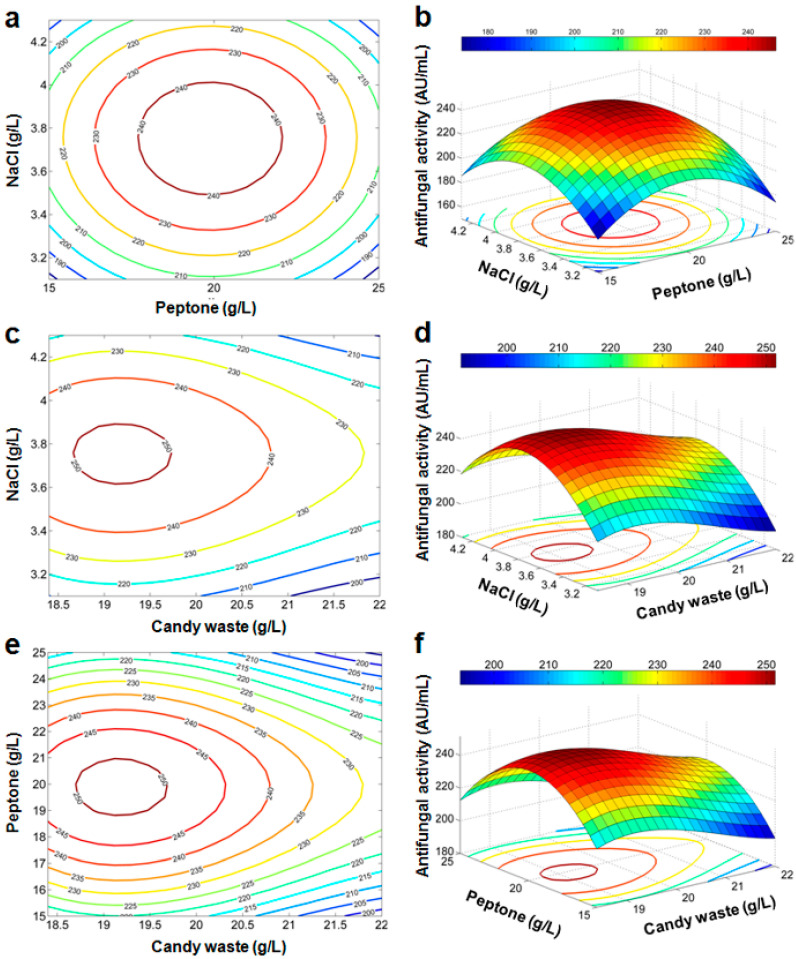
Contour plots (**a**,**c**,**e**) and response surface curves (**b**,**d**,**f**) predicted by the retained polynomial model.

**Figure 4:**
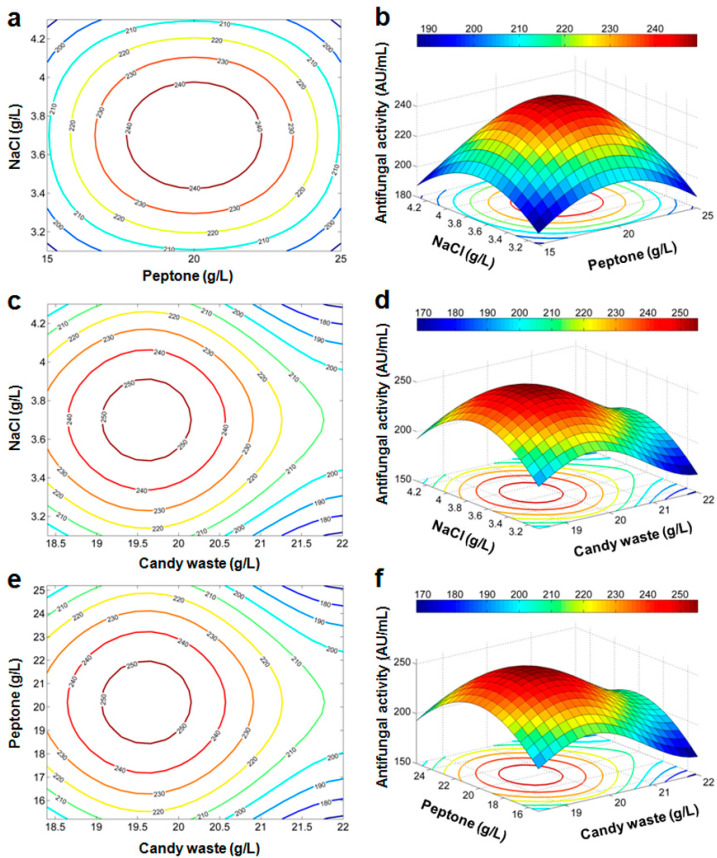
Contour plots (**a**,**c**,**e**) and response surface curves (**b**,**d**,**f**) predicted by the trigonometric model.

For a graphical illustration of the regression Equations (Equations (5) and (6)), contour plots and 3D response surfaces are represented (Figure 3 and Figure 4). In these plots, one of the three factors is set to its mean level (its value at the central point in the design space) while the two others are varied. Contour lines are concentric curves with increasing levels. Consequently, the antifungal activity should reach its maximal value for a specific combination of factors inside the considered domain.

##### Optimization of Production Conditions of the Antifungal Activity

For optimization of the antifungal activity, we carry a triple loop search sweeping the possible values of (*X_1_*, *X_2_*, *X_3_*) in the domain (18.4; 22) × (15; 25) × (3.1; 4.3) (g/L) with a step of 0.01 (g/L). The polynomial model (Equation (5)) yields a maximum of antifungal activity of 251.9 (AU/mL) for *X_1_* = 19.17, *X_2_* = 19.88, and *X_3_* = 3.75 (g/L). Using the trigonometric model (Equation (6)), the maximal value of antifungal activity of 255.5 (AU/mL) is achieved for *X_1_* = 19.61, *X_2_* = 20, and *X_3_* = 3.7 (g/L). The optimized values with both models are very close to the center point of CCD, and only a slight alteration of *X_1_* should be applied.

## 4. Conclusions

PBD, SAM, and CCD were applied to maximize the antifungal production by *B. amyloliquefaciens* BLB369. Candy waste, peptone, and NaCl, the most significant among seven factors, were used for regression in CCD. Regression analysis showed the supremacy of a new trigonometric model over the usually used polynomial model, which may encourage using the trigonometric model in CDD. The residuals plot showed the adequacy of both models (unbiased). Moreover, contour plots and numerical optimization revealed a maximal activity for a point close to the CCD center. This low-cost medium could be investigated to further optimize BLB369 and similar strains for industrial and agricultural applications to control fungal diseases.

## Figures and Tables

**Table 1:** The Plackett–Burman experiments design matrix with factors given in coded levels and biofungicide production values.

Run	*A*: Candy Waste	*B*: Fish Extract	*C*: Peptone	*D*: Yeast Extract	*E*: NaCl	*F*: MgSO_4_	*G*: MnSO_4_	Antifungal Activity (AU/mL)
**1**	+1	−1	−1	+1	−1	+1	+1	75
**2**	+1	+1	−1	−1	+1	−1	+1	75
**3**	+1	+1	+1	−1	−1	+1	−1	150
**4**	−1	+1	+1	+1	−1	−1	+1	125
**5**	+1	−1	+1	+1	+1	−1	−1	175
**6**	−1	+1	−1	+1	+1	+1	−1	62.5
**7**	−1	−1	+1	−1	+1	+1	+1	150
**8**	−1	−1	−1	−1	−1	−1	−1	0

Variables in real values (g/L): *A* (10, 20), *B* (0, 16), *C* (0, 10), *D* (0, 5), *E* (0, 4), *F* (0, 0.5), and *G* (0, 0.006).

**Table 2:** Statistical analysis of factors using Plackett–Burman design.

Coefficient	Value	*p*-Value	Significance
*α_0_*	101.563	3.49 × 10^−12^	***
*α_1_*	17.188	4.15 × 10^−6^	***
*α_2_*	1.563	0.347	
*α_3_*	48.438	1.27 × 10^−9^	***
*α_4_*	7.813	1.05 × 10^−3^	**
*α_5_*	14.063	1.85 × 10^−5^	***
*α_6_*	7.813	1.05 × 10^−3^	**
*α_7_*	4.688	0.017	*

*** Significance level 99.9%; ** significance level 99%; * significance level 95%. *R^2^* = 0.9935; *AR^2^* = 0.9878.

**Table 5:** Regression models: regression coefficients, their significance, and some statistical parameters.

Model	Term	Coefficient	*p*-Value	Significance
**Polynomial model ^#^**	Intercept	−2050.5	0.03693	*
	*X_1_*	64.534	0.2974	
	*X_2_*	57.418	0.0151	*
	*X_3_*	573	0.0062	**
	*X_1_^2^*	−2.5428	0.0703	
	*X_2_^2^*	−1.3295	9.75 × 10^−6^	***
	*X_3_^2^*	−92.33	9.75 × 10^−6^	***
	*X_1_ X_2_*	0.3472	0.6739	
	*X_1_ X_3_*	8.6806	0.2227	
	*X_2_ X_3_*	−3.125	0.2227	
**Retained polynomial model after applying the stepwise technique ^§^**	Intercept	−12312	0.0108	*
	*X_1_*	1623.8	0.0211	*
	*X_2_*	52.849	7.74 × 10^−7^	***
	*X_3_*	351.1	7.21 × 10^−7^	***
	*X_1_^2^*	−78.306	0.0245	*
	*X_2_^2^*	−1.329	6.38 × 10^−7^	***
	*X_1_^3^*	1.2503	0.0285	*
	*X_3_^3^*	−8.3034	6.34 × 10^−7^	***
**Trigonometric model ^£^**	Intercept	144.52	3.1 × 10^−19^	***
	$\cos\left({\bar{X}}_{2}\right)\;\cos\left({\bar{X}}_{3}\right)$	87.202	6.6 × 10^−13^	***
	$\cos\left(5.5358-2.2737{\bar{X}}_{1}\right)$	23.775	2.9 × 10^−6^	***

*** Significance level 99.9%; ** significance level 99%; * significance level 95%. ^#^ *R^2^* = 0.920; *AR^2^* = 0.848; *AICc* = 208. ^§^ *R^2^* = 0.928; *AR^2^* = 0.887; *AICc* = 190. ^£^ *R^2^* = 0.962; *AR^2^* = 0.957; *AICc* = 161.

**Table 6:** ANOVA for significance of regression and lack of fit of the retained polynomial model and the trigonometric model.

Model		Mean of Square	*F*-Value	*p*-Value
**Retained polynomial model**	**Total**	2728.6		
	**Model**	6876.2	22.24	5.717 × 10^−6^
	**Residual**	309.17		
	**Lack of fit**	530.01	Inf	0
	**Pure error**	0		
**Trigonometric model**	**Total**	2728.6		
	**Model**	24926	212.71	9.32 × 10^−13^
	**Residual**	117.18		
	**Lack of fit**	175.77	2.0624	0.1413
	**Pure error**	85.227		
